# Synopsis of Proceedings Twenty-third Annual Meeting of the Texas State Medical Association at Tyler

**Published:** 1892-05

**Authors:** 


### Society Notes.

### TEXAS STATE MEDICAL ASSOCIATION.—SYNOPSIS OF PROCEEDINGS.

The 23rd Annual Convention of the Texas State Medical Association was held at Tyler, Texas, on the 26th, 27th and 28th ult.

The meeting was called to order with about fifty present. President Dr. W. H. Wilkes, of Waco, in the chair. After prayer by Rev. Mr. Turner of the Episcopal church, Dr. C. A. Smith, chairman of Committee of Arrangements, reported the action of his committee (details of which, for the most part, were given in our last issue).

Inasmuch as Tyler had lost two of its three hotels by fire recently, there was not sufficient accommodation for even the comparatively small number of delegates in attendance the first day, and the committee announced that four hundred citizens of Tyler had thrown open their doors and would each receive a share of the delegates as guests.

The mayor, Mr. Long, delivered an address of welcome, and "tendered the freedom of the city." Dr. H. L. Tate, a physician of Smith county, near Tyler, delivered an address of welcome on behalf of the medical profession of Tyler and Smith county. Ex-Governor Hubbard was introduced and also welcomed the doctors in a pleasing and most complimentary speech. He described the "progress of medicine" in fine style, and paid a handsome tribute to the Association; also to the Texas Medical College, and quoting from the address of Dr. Paine, Dean of said school, said, that more money was carried out of Texas each year by medical students attending lectures than would be required to build the finest college building in the world, and urged Texas to educate her doctors at home.

The chairman of the Committee of Arrangements then read the programme for the social part of the occasion, which consisted of a series of receptions at the residences of leading citizens, carriage drives around the city, and a rousing banquet for the third evening, after the President's address.

The Secretary and Treasurer read their respective reports, and the Secretary read the report of the Publishing Committee. Reports referred to appropriate committees and approved as read.

Resident Wilkes then read the ''annual message and recommendations.'' It opened with mention of those who had died since last meeting, Drs. W. W. Reeves, W. T. Strain, T. M. Stone and T. S. Burke, paying a glowing tribute to their memory.

The President called attention in his message to the action of the American Medical Association at its last meeting, in changing, by *viva voce* vote, the place of meeting (Hot Springs, Ark.) chosen by its nominating committee, and said the Texas State Association should enter its protest against such action; called attention to the Pan-American Medical Congress and asked that the Association take such action as it thought proper; spoke of the memorial to Congress to create a Bureau of Public Health with a Medical Secretary or other executive officer in the Cabinet, and asked that the Association endorse said memorial, etc.

The President's message and recommendations were referred to a committee, Dr. E. P. Becton, chairman. The committee reported in accordance with the President's suggestions, in effect protesting very forcibly against the precedent of the A. M. A. in setting aside the action of its nominating committee, as dangerous and unconstitutional, and declared, if followed, it would be destructive of the fundamental principles of the organic law of the Association, and a repetition of it would destroy the usefulness of that organization. But one of the worst results, the committee said, of the action of the body in this particular case in changing the place of meeting from Hot Springs, Ark., to Detroit, Mich., was that Detroit being further off from Texas and the South, and more inaccessible, many Southern, and especially Texas, delegates would be prevented from attending the meeting, much to the detriment of the Association. With regard to the memorial to Congress concerning a Secretary of Public

Health the committee reported a series of resolutions endorsing said memorial and urging upon Congress an earnest consideration of the claims set up in behalf of the public health; reciting that the interests of the public health should be paramount to any other interest in the economy of an enlightened government, and that in America said interests had received insufficient recognition and attention; that they were of such magnitude and importance as to demand a separate department, such at least as is devoted to agriculture and live-stock, and an executive officer to rank as a minister, etc.

The report of the committee was adopted, and especially the resolutions just referred to, with enthusiastic applause.

The President recommended a revision of the by-laws whereby the function, or rather the powers, of the Judicial Council, with regard to trials and punishments should be more clearly defined. No action was had on this point.

The afternoon session of the first day was devoted to Section work.

SECOND DAY—MORNING SESSION, APRIL 27.

Quite an accession to the ranks came in on the night of the first day, swelling the number perhaps to two hundred.

The Section on Surgery being called, Dr. E. P. Becton was requested to act as chairman in place of Chairman C. H. Wilkinson, who was absent, and Dr. Osborn acted as Secretary. Under this Section several papers were read, and referred to the Publishing Committee. [Titles given in our last issue, before the meeting.]

Dr. Samuel E. Milliken, a young physician connected with the New York Polyclinic, was introduced, and, by request, exhibited some colored drawings demonstrating Bassini's method of operating for the radical cure of hernia. Dr. Milliken having been born in Texas, and being a self-made man; having studied medicine and begun practice near Dallas, and by force of his own labors risen to a position of prominence in the great medical center, New York, was made much of. He was listened to with marked attention as he delivered a creditable lecture, and at its close he was made the recipient of a vote of thanks, reciting the facts above mentioned, and closing with the declaration that Texas and this Association are proud of Dr. Milliken, as a Texas boy. He was made an honorary member.

The Judicial Council was called, and adjourned to the court room for deliberation.

Dr. J. W. Carhart read a paper on the removal of a tumor from the brain by a surgeon in St. Louis.

Dr. Irvin Pope reported a case of supra-pubic operation in a patient of advanced age. This paper elicited some interesting discussion.

Dr. Daniel, by request, read a paper by Dr. J. Cummings, of Austin, on "Some practical work in surgical treatment of the liver." Referred, as were all the others, to Publishing Committee.

Adjourned to 2:30 p. m.

AFTERNOON SESSION—SECOND DAY, WEDNESDAY, APRIL 27.

Dr. Becton in the chair as chairman Section on Surgery. Dr. Wilkinson being absent, the chairman's address on Surgery was read by the Secretary, Dr. West. It was very voluminous, and pronounced able. It was entitled "The Present Status of Surgery," and was a review of the progress—a favorite subject, even though pretty threadbare.

First Vice President P. C. Coleman in the chair.

Section on Practice called; chairman, Dr. T. J. Bell. Dr. J. P. Tucker read a paper on typhlitis. Dr. Bell's address in medicine was one of the ablest and best papers read at the meeting.

Dr. H. A. West read a paper on miliary tuberculosis. Papers referred to Publishing Committee.

The following gentlemen were proposed for honorary membership, and all were duly elected: Drs. Jos. Price, Philadelphia; L. F. McMurtry, Louisville; Jno. C. Cardwell, Boston; W. Gillian Thompson, New York; J. M. Keller, Hot Springs, Ark., G. Frank Lydston, Chicago, and I. N. Love, of St. Louis.

Dr. Keller was the only one of the above gentlemen present, and by vote he was invited to a seat in convention, and to participate in the discussions.

The Judicial Council reported upon the charges against Dr. Jas. Kennedy, of San Antonio,—violation of the code of ethics; specification—putting a paragraph in the newspaper to the effect that he had performed a surgical operation on a person (no particulars). Dr. Kennedy was exonorated.

New members admitted on report of Judicial Council: Drs. W. P. Collin, J. H. Earps, Clay Johnson, Geo. W. Benton, T.

B. Bass, John T. Motley, C. J. Holt, W. P. White, M. M. de la Garza, Albert G. Branlet, H. L. Hilgartner, Wm. H. Baldinger, E. K. McKenzie, B. E. Selman, C. O. Matthews, D. P. Richardson, F. W. Swindall, W. H. Monday, W. B. Newland, E. S. Collier, J. S. L. Gray, D. G. Shirley, W. W. Pugh, S. L. Butler, H. L. Tate, M. C. Lynch, M. Smith, M. H. Cravens, E. D. Capps.

Dr. Q. C. Smith offered a resolution that the Association request all railroads in Texas to permit physicians called to go over any line on professional duty to travel on freight or other trains at their own risk, by paying full fare.

On motion, it was *laid upon the table!*

Dr. Smith offered a resolution to rescind the ordinance setting aside $300 as compensation to the Publishing Committee each year for editing and publishing the volume of Transactions.

Referred to a committee of five, Dr. W. P. Burts, chairman, and committee reported adversely,—to lay upon the table.

Dr. Smith offered another resolution to revise the by-laws relating to the election of officers, and proposing that said election be by the Association in convention, and not by nominating committee. Motion was tabled.

Adjourned to 9:30 Thursday a. m., 28th.

THIRD DAY, MORNING SESSION, WEDNESDAY, APRIL 28.

After preliminaries, Section on Obstetrics called; officers absent and Section passed; no papers in this important Section, we believe.

Section on Medical Jurisprudence and Psychology was called. Dr. W. W. Reeves, the chairman of this Section, it will be remembered, was killed in Austin in December, 1891, and Dr. B. F. Church, of Terrell, First Assistant Physician to the North Texas Hospital for the Insane, was called to preside.

Dr. Kennedy, of Hillsboro, read a paper entitled, "An Inebriate Asylum for Texas." This paper was a strong prohibition document, and would have made good campaign literature in '88. It provoked much discussion—not so much as to its merits or features as to what should be done with it. As there was attached to it and read a draft of a bill to create such asylum, and prescribe its management, a member moved it be referred to the legislature—lost. Another wanted the paper referred to the Publishing Committee, and the bill to the waste basket. Another *vice versa*, etc. Motions were made, and substitutes offered, till Dr. Church got "rattled," and asked an older hand than he to take hold and untangle the "parliamentary knot." Dr. Wilkes, the President, relieved the modest young doctor, and got rid of Dr. Kennedy's paper and bill; they were referred to Publishing Committee, which body, as is known, has discretionary powers with regard to all papers referred to them.

Resignation of Dr. J. G. Adams read and accepted.

Section on Gynecology called; no papers.

The hour (11 a. m., third day) for "Memorial Services" having arrived, the Association entered upon the service by prayer and appropriate addresses. Dr. E. P. Becton, the eagle orator of the Association, was called for, and he delivered one of those beautiful *extempore* speeches for which he is famous, paying a handsome tribute to the merits and memories of the departed brethren; it was poetry in prose, and most touching in tone and sentiment. To the qualities of head and heart of Dr. Reeves, especially, he paid a glowing tribute—an eulogy, indeed, upon his life and character.

After the close of memorial service, when the report of Committee on President's Recommendations was read (see first day), Dr. J. W. Carhart offered a resolution endorsing the scheme of the Pan-American Medical Congress, to be held in '93, at the time of holding the Columbian Exposition. Adopted.

Section on State Medicine and Public Hygiene was called, Dr. J. L. Cunningham, Chairman, and Dr. J. D. Osborn, Secretary. Dr. Cunningham read an able paper, in which he outlined a system for the carrying out of the present law for the protection of Texas from infectious diseases, etc.

Recess of ten minutes to organize the Nominating Committee. Committee retired for work. Election of officers, etc.

Adjourned to 2:30 p. m.

THIRD DAY, AFTERNOON SESSION.

Called to order at 2:30 p. m. President Wilkes in the chair. The Nominating Committee reported the officers for the next year as follows:

President—J. D. Osborn, Cleburne, Texas.
First Vice-President—T. J. Bell, Tyler, Texas.
Second Vice-President—F. M. Pitts, Jr., Hubbard, Texas.
Third Vice-President—T. J. Bennett, Austin, Texas.—[Editor of the TEXAS SANITARIAN.]

[A list of the officers of Sections was not secured. It is well enough, perhaps, for those elected heretofore to such offices, with few exceptions, have failed to fill the positions, discharge the duties, or attend the meetings, and it matters little who are elected. New officers for the most part have to be appointed when the meeting takes place. Such has been the case.]

The newly elected officers were inducted into office, each conducted to the stand by a select committee, and each made a graceful acknowledgment of the honor.

Galveston was reported as the choice of the Nominating Committee for next meeting, and inasmuch as the usual time of holding the annual meeting,—fourth Tuesday in April,—conflicts with the closing exercises of the Medical College, whose term closes the last week in April, it was resolved that the next meeting be held one week later, to-wit: first Tuesday in May, 1893.

President-elect J. D. Osborn took the gavel, and business was proceeded with.

Little else of importance was done after the report of the Nominating Committee. The greater part of the crowd dispersed, and passed the afternoon riding around the city, under escort of the hospitable Committee of Arrangements, viewing the buildings and grounds of the beautiful little city, and being shown her public enterprises.

At 8:30 p. m. the opera house was filled with fair women and brave men, or we may say brave men and brave women; for a terrible storm was gathering, dark and threatening enough to deter any but the bravest from venturing out; but as expectation was high, and a great intellectual treat expected in the address of President Wilkes, so famed for originality and wit, everybody turned out to hear him; and scarcely had the crowd settled in their seats before the storm broke with great fury, great hailstones falling upon the roof with deafening roar.

In the midst of the address,—a fine one,—an old member, a patriarch in the tribe medical, rose in his seat and said: "We can't hear you; sit down till the storm is over."

After the address Hon. Horace Chilton spoke, and also Dr. Becton.

President J. D. Osborn declared the Texas State Medical Association adjourned till first week in May, 1893.

Some of the members—well—most of them went to the banquet hall, where a "feast"—no—where a magnificent supper'

with toast accompaniments, was served, and speech making was indulged in "till the storm rolled by"—about 2 a. m.

No wine; no ladies present; dry (?) toast(s) and wet weather —rather a damper on the zeal of the energetic Reception Committee, who had done their whole duty, like the splendid gentlemen they are. Much credit is due them; and they assuredly have the thanks of all present, as well as have the hospitable citizens who so cordially took us in, and so graciously and agreeably administered to the wants of the delegates.

